# Artificial Intelligence for Predicting Length of Stay for General Surgery Patients: A Systematic Review

**DOI:** 10.1002/wjs.70570

**Published:** 2026-09-17

**Authors:** Joshua Blum, Nomiki Glynatsis, Brieanna Hill, James Montgomery, Richard Turner

**Affiliations:** ^1^ Tasmanian School of Medicine University of Tasmania Hobart Australia; ^2^ Department of General Surgery Royal Hobart Hospital Hobart Australia; ^3^ School of Engineering and ICT University of Tasmania Hobart Australia

**Keywords:** artificial intelligence, machine learning, optimisation, patient flow, surgery

## Abstract

**Background:**

Length of stay (LoS) prediction is vital for optimizing surgical patient flow and resource allocation. Artificial intelligence (AI) and machine learning (ML) models may enhance LoS prediction, yet their validity and clinical utility in General Surgery remain unclear.

**Methods:**

Embase, Medline, Scopus, and Web of Science were systematically searched to June 2025. Studies employing AI or ML to predict LoS or discharge timing in adult General Surgery cohorts were included. Data on model development, predictor and target variables, performance, and validation were extracted. Risk of bias was appraised using PROBAST + AI. A checklist was developed to guide future LoS‐prediction research.

**Results:**

Nine of 4115 screened records met inclusion criteria. Classifier models with low overall risk of bias achieved moderate discrimination (AUROC 0.71–0.83), whereas regression models performed poorly (RMSE 4.6–12.7 days). Methodological weaknesses were frequent, including data leakage, temporally invalid predictors, biased patient selection, limited external validation, and incomplete performance reporting. Important predictors commonly included ASA score, age and operative duration; psychosocial and home‐support features were rarely considered. Outcome definitions and intended applications varied substantially, limiting direct comparison of performance. Models predicting discharge within short, dynamically reassessed time horizons may achieve greater accuracy, but offer limited capacity for longer‐term bed forecasting. No included study benchmarked ML or AI predictions against usual clinician LoS estimates. Low‐bias models were retrospective and generally internally validated, so performance in prospective or external cohorts remains unknown. More complex neural network models did not consistently outperform simpler methods. This review identifies recurring barriers to prospective clinical application for routine real‐world use.

**Conclusion:**

AI and ML models can moderately predict LoS for General Surgery patients, but generalizability is limited by methodological bias, incomplete reporting, and non‐standard outcome definitions. Current models appear more suited to hospital‐wide forecasting than bedside decision support. Future development should use prospectively available predictors, clinically meaningful outcomes, transparent performance reporting, and external validation. The proposed framework highlights strategies to avoid common methodological flaws in models published to date.

## Graphical abstract

Length of stay (LoS) prediction is vital for optimizing surgical patient flow and resource allocation. Artificial intelligence (AI) and machine learning (ML) models may enhance LoS prediction, yet their validity and clinical utility in General Surgery remain unclear. AI and ML models can moderately predict LoS for General Surgery patients, but generalizability is limited by methodological bias, incomplete reporting and non‐standard outcome definitions. Current models appear more suited to hospital‐wide forecasting than bedside decision support. Future development should use prospectively available predictors, clinically meaningful outcomes, transparent performance reporting and external validation. The proposed framework highlights strategies to avoid common methodological flaws in models published to date.

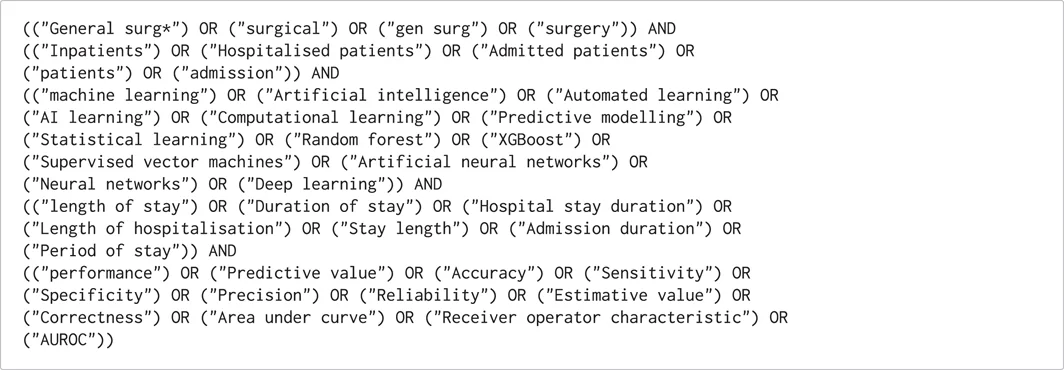

## 1 Introduction

Length of stay (LoS) is a vital measure that is often used as a surrogate for admission efficiency. Beyond its use as a performance indicator, the ability to accurately predict a given patient's anticipated LoS allows hospital bed managers to forecast bed occupancy, which informs critical downstream decisions such as staff rostering, elective case scheduling, and resource allocation [1]. Furthermore, reduced LoS is associated with reduced rates of in‐hospital complications, increased patient satisfaction and better patient outcomes [2, 3]. Thus far, implementation of formalized LoS predictive models beyond research settings has been limited, and many healthcare systems rely on ad hoc predictive measures, such as clinician estimation, to help inform bed occupancy forecasting [4].

Artificial intelligence (AI) and machine learning (ML) techniques have emerged as a promising tool, able to model non‐linear relationships in data to predict a variety of outcomes. Specifically, supervised AI and ML models are trained on labeled data in order to learn the relationships between predictor variables and the target variable, such that they can then be applied to unlabeled data in order to generate predictions about this data [5]. This is useful for complex, interconnected systems such as hospital wards and operating theaters, where small variations in input data can compound into larger, seemingly unpredictable outcomes that linear or rule‐based models struggle to capture [6, 7]. General Surgery cohorts represent a heterogenous patient population with varying degrees of pathology, disability, and care requirements [8]. As such, models that consider a multitude of variables with often non‐linear interactions are suitable for predicting outcomes in these patients.

Despite growing interest in this field, the reliability and clinical utility of these models in General Surgery remains uncertain, as do the steps to implement them into clinical workflows. Although previous reviews have examined LoS prediction more broadly across healthcare systems [2], there is no focused evaluation of General Surgery patient populations. Furthermore, no review of this type has applied the recently updated PROBAST + AI [9] framework to assess risk of bias. This review seeks to evaluate the literature reporting AI and ML model performance in predicting LoS in General Surgery patient cohorts, as well as to provide direction for future projects in this domain.

## 2 Methods

### 2.1 Data Sources and Search Strategy

A systematic search of Embase, Medline, Scopus, and Web of Science databases was employed. Full text articles published in peer‐reviewed journals in English up until June 2025 were included. Two reviewers independently screened all records for eligibility, with discrepancies resolved through consultation with a third reviewer. Search terms are listed in Figure 1. In cases of reporting inconsistency, authors were contacted for clarification. This study is reported in accordance with PRISMA guidelines and is PROSPERO registered (ID CRD420251151694).

**FIGURE 1:**
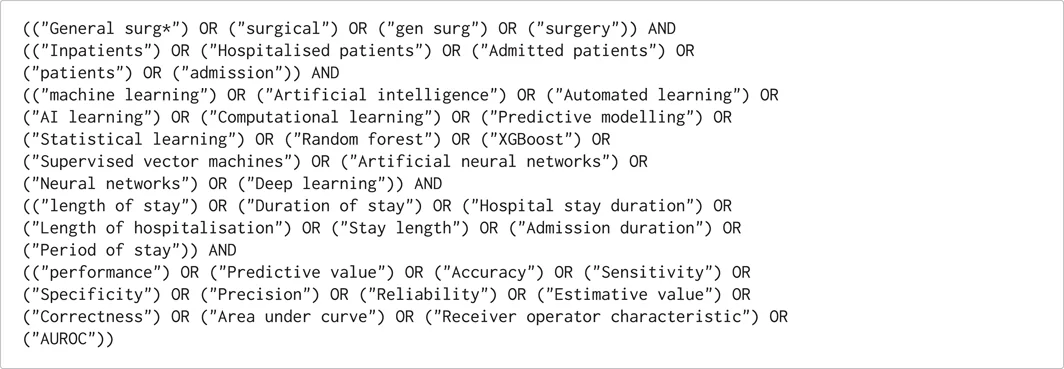
Search terms for database search. Detailed search terms applied to the “all text” query of each of the utilized databases.

### 2.2 Study Eligibility Criteria

We included published studies that evaluated ML and AI‐based models utilized to predict the LoS or timing of discharge in General Surgery patients. No exclusion criteria were developed on the basis of the ML techniques employed. The primary outcome was each model's accuracy in predicting LoS or prospective estimation of discharge. Papers that also reported model performance for outcomes unrelated to LoS were included, but this review does not assess model performance for these outcomes.

### 2.3 Data Extraction

Data of interest included the ML techniques used, variables used to train models, data sources, timing of data collection as well as timing of model application within an admission. Performance was represented by area under receiver operator curve (AUROC) scores, accuracy, and sensitivity/specificity for classification tasks, and mean absolute error (MAE), root mean squared error (RMSE) mean absolute percentage error (MAPE) for regression tasks. If published, predictor variable importance was reviewed. Meta‐analysis was not possible given the heterogeneity of patient cohorts and ML/AI techniques used.

### 2.4 Risk of Bias Assessment

Included studies were appraised using the Prediction Model Risk of Bias Assessment Tool + AI (PROBAST + AI) [9] and this appraisal visualized using the “robvis” tool [10]. PROBAST + AI is used to assess studies across four domains: the population/setting that the model is applied to; the included predictor variables; the output variables; and the ML‐specific analysis that has been performed [9].

## 3 Results

4115 articles were screened for eligibility; 35 required full text review of which 9 were eligible for inclusion [11, 12, 13, 14, 15, 16, 17, 18, 19]. This process is displayed in Figure 2.

**FIGURE 2:**
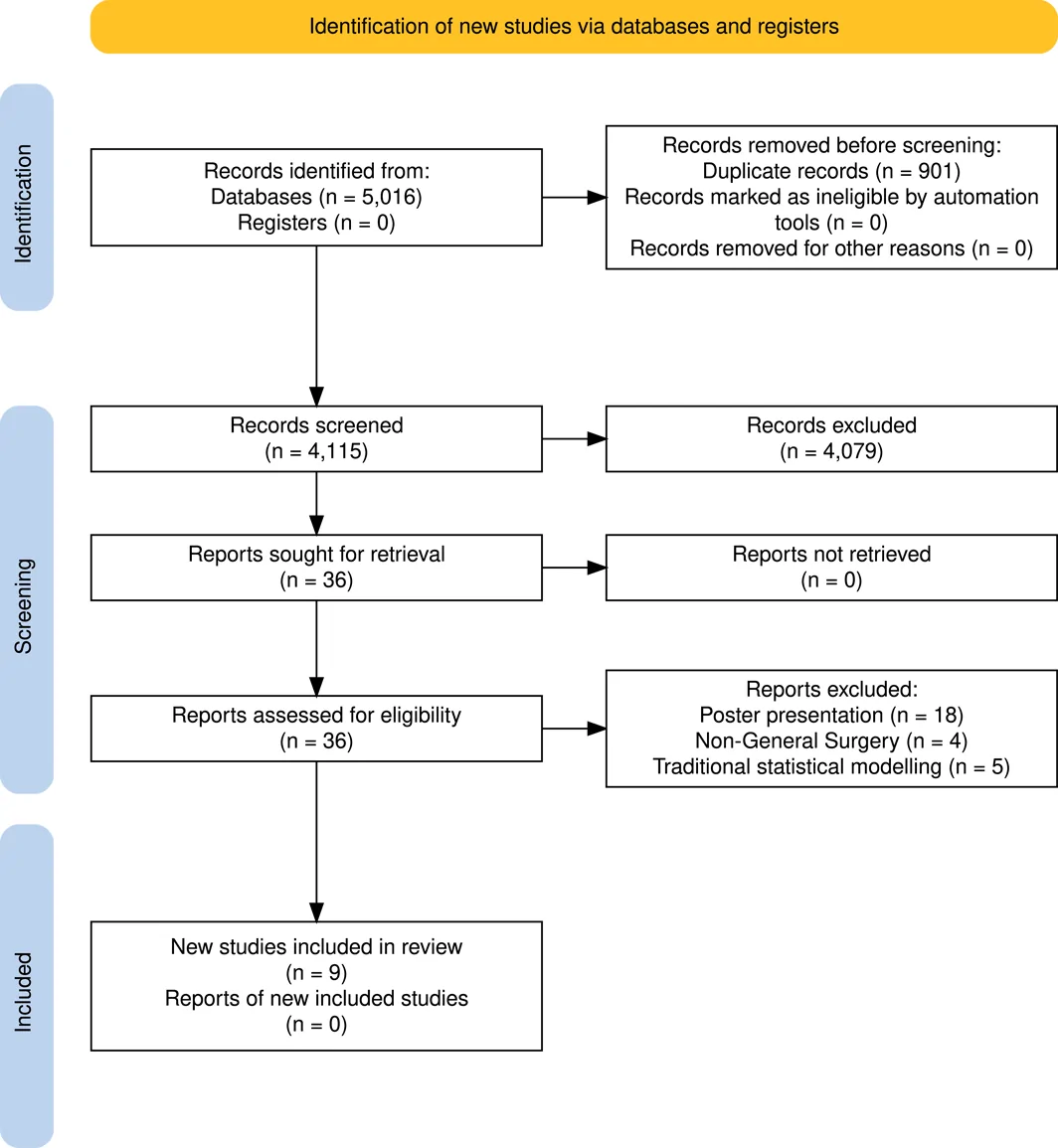
PRISMA flow diagram demonstrating study selection. PRISMA flow diagram demonstrating study selection filters and drop out of studies throughout filter application.

### 3.1 Study Characteristics

Detailed study characteristics are represented in Table 1.

**TABLE 1:** Study characteristics.

Author/Year/Country	*n* =	Setting	Subspecialty	Prediction outcome
Median‐based classifiers				
Anania et al. 2024, Italy [11]	2012	Elective	Colorectal	LoS > median in patients post laparoscopic right hemicolectomy
Capretti et al. 2021, Italy [12]	100	Elective	HPB	LoS > median in post pancreatoduodenectomy
Wen et al. 2023, China [18]	83	Elective	Colorectal	LoS > median in patients undergoing colorectal cancer resection
Xu et al. 2022, USA [19]	42,209	Elective	Colorectal	LoS > median/regression output for patients undergoing elective surgery
Set duration/resource‐based classifiers				
Dudurych et al. 2020, Int [13]	1147	Elective	Colorectal	LoS > 14 days for post‐operative pelvic exenteration patients
Jo et al. 2021, South Korea [14]	42,751	Elective	NOS	Prolonged LoS as defined by study methods in surgical cancer patients
Jones et al. 2025, Australia [15]	5195	Emergency	NOS	LoS > 7 days/regression output for patients undergoing emergency laparotomy
Scala et al. 2023, Italy [17]	2352	Elective	HPB	LoS > 8 days/regression output
Discharge within a fixed timeframe classifiers				
Kovoor et al. 2023, Australia [16]	14,690	Elective and emergency	NOS	Discharge within 12 or 24 hours

Abbreviations: HPB = hepatobiliary, LoS = length of stay, NOS = not otherwise specified.

### 3.2 Risk of Bias

The risk of bias visualization is displayed in Figure 3. Three studies had a low overall risk of bias, two were unclear or had a moderate risk, and four possessed a high risk. The domains most commonly contributing to an increased risk of bias were the predictor variables selection processes and the ML/AI analysis performed.

**FIGURE 3:**
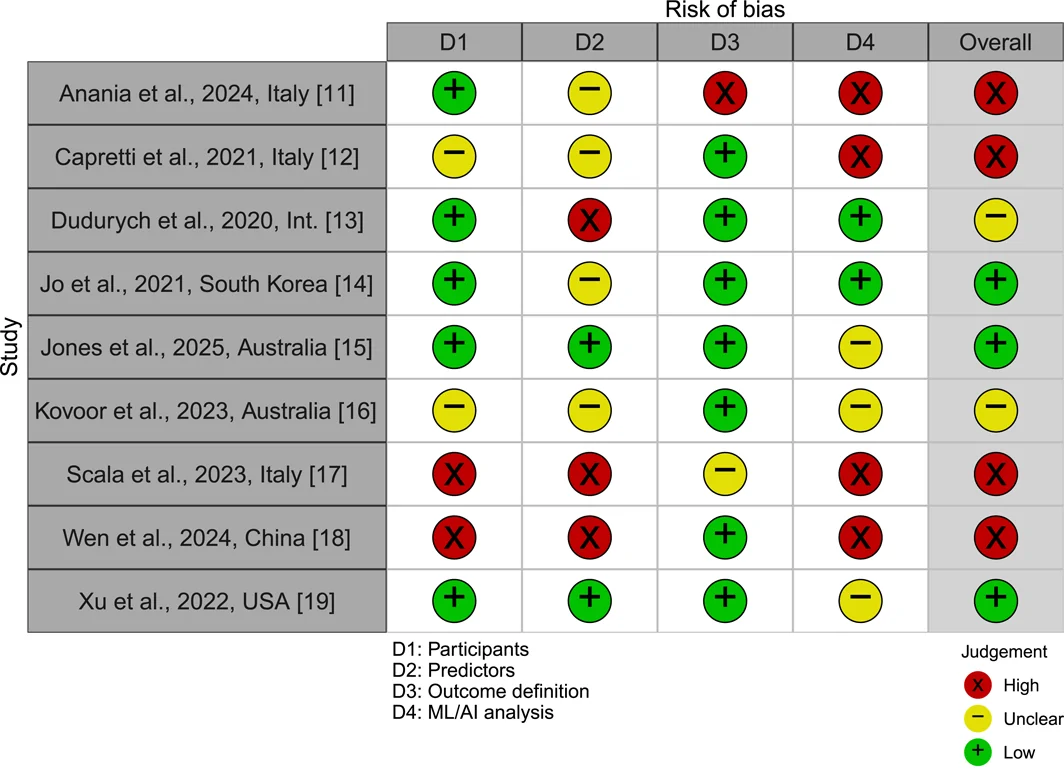
Risk of bias assessment visualization. PROBAST + AI risk of bias assessment visualization using “robvis” webapp.

#### 3.2.1 Patient Selection Bias

Most papers demonstrated a low risk of bias regarding patient selection. One study sourced data on an international multicentre scale [13], two others from a national multicentre scale [11, 15], and the rest from single centers. Of the single center datasets, four had a comparatively large volume of included patients [14, 16, 17, 19] and two a small volume of patients [12, 18].

Three papers raised concerns of selection bias in their eligibility criteria; Scala et al. [17] and Wen et al. [18] excluded patients who were deemed “more complex” or “higher risk”, thus restricting the applicability of the reported models to well patients, whose LoS is likely inherently easier to predict. These papers were appraised as having a high risk of bias stemming from patient selection. Kovoor et al. [16] excluded patients who were readmitted within 30 days of discharge. Given this paper's aim to predict patients' suitability for discharge [16], excluding patients who failed discharge likely excludes the patient population for whom accurately predicting discharge timing is most difficult. This paper was therefore deemed as having a moderate risk of bias in this regard.

#### 3.2.2 Predictor Variable Bias

Three studies demonstrated moderate risk of bias associated with predictor variable handling during pre‐processing of the data [11, 12, 14]. Anania et al. [11] contained a reporting error that introduces concerns regarding data‐handling. They reported that 93.5% of their derivation dataset received an intraoperative blood transfusion [11]. This is compared with their validation dataset, wherein only 3.55% of patients required a transfusion. This is likely due to inadvertent class reversal for this variable [20]. If the reversal occurred prior to training models, this would have negatively impacted performance. This study also reports that blood transfusion was more important for performance than patients' American Society of Anesthesiologists (ASA) score, body‐mass index (BMI), and comorbidities [11]. This paper has a moderate risk of bias due to this mistake.

Capretti et al. [12] and Jo et al. [14] demonstrated a moderate risk of bias due to potential data leakage during variable pre‐processing. Both papers report that standardization and imputation of the data occurred prior to splitting the data into training and testing datasets [12, 14]. This constitutes data leakage from the testing into the training dataset as these transformations implicitly depend on data that the model should not have seen during testing. An accepted approach is to standardize/impute the training dataset and then apply the generated scaler to the testing/validation datasets, as performed by Kovoor et al. [16] and Dudurych et al. [13].

Three papers had a high risk of bias related to temporal inconsistencies in their predictor variables [13, 17, 18]. Dudurych et al. [13] included variables that could not meaningfully be included in prospectively applied models. These include 30‐day readmission, complication and mortality rates, as well as pathologic evaluation of the cancer resection [13]. As these variables are made available after the patient has already discharged, their inclusion in a model aiming to predict LoS introduces a high degree of bias. Scala et al. [17] include pre‐operative LoS as a predictor variable, which constitutes severe data leakage given total and pre‐operative LoS are inextricable. Wen et al. [18] introduce a high level of bias by including variables that could not be known prospectively, such as walking distance 3 days post‐operatively and complication rates.

The two papers that had a low risk of bias associated with predictor variable selection clearly specified the timing of variable collection and model application [15, 19]. Jones et al. [15] applied models at two time points; pre‐operatively and immediately post‐operatively. They excluded any incomplete records, which minimizes bias introduced by imputation but limits generalizability [15].

#### 3.2.3 Outcome and Result Bias

There were diverse definitions of prolonged LoS across papers. Four papers defined prolonged LoS as staying longer than the median LoS [11, 12, 13, 18]. Two papers selected arbitrary cut‐offs to categorize patients by; Jones et al. [15] dichotomized the patient cohort into those who stayed less than or longer than 1 week, and Scala et al. [17] categorized patients into short, medium, and long stay “bins.” Although somewhat arbitrary, these methods are unlikely to introduce bias to the studies. Scala et al.’s [17] categorization developed significantly imbalanced classes, possibly inflating model performance via the accuracy paradox [21].

Three papers' definitions were clinically relevant. Jo et al. [14] separated patients into their cancer subtypes, then ordered their patients by LoS. They then classified those who's cumulative LoS accounted for 50% of total bed days, as being the positive class. This definition of prolonged LoS is inextricably linked to bed utilisation and is a non‐arbitrary method of identifying patients who occupy most clinical resources.

Rather than defining LoS, Kovoor et al. [16] predicted whether a patient would be discharged within 12 or 24 hours. This outcome is notable in that it is agnostic to admission length, instead framing discharge prediction as a daily reassessment. However, clinical readiness for discharge is likely distinct from exact timing of discharge, as the latter is often influenced by logistic factors, unrelated to the specific patient's medical suitability to leave hospital. This might introduce an outcome misclassification relative to the true construct when considering clinical readiness for discharge but is less likely to impact applications wherein bed availability is concerned.

A two‐stage approach to modelling prolonged LoS is described by Xu et al. [19], who developed a classifier model to predict LoS less than or greater than 1 week, and a second regressor model to predict LoS in days for the longer staying patients. This is a clinically meaningful outcome as it avoids the arbitrariness of a fixed cutoff and instead models the heterogeneity of prolonged admissions by providing a regression output for these patients.

Anania et al. [11] developed a regression model predicting LoS in days. However, they defined a “correct” prediction as any estimate within 3 days of the true LoS [11]. This gave the model a 7‐day margin of error for correct predictions, which probably artificially bolstered model accuracy and is unlikely to provide meaningful clinical insight.

#### 3.2.4 Machine Learning Analysis Bias

ML methods were often applied without adherence to accepted analytical standards and as such many papers featured a high risk of bias in this domain. Scala et al. [17] and Wen et al. [18] used highly dimensional predictor spaces, applied to comparatively small datasets. This increases the risk of overfitting the models, wherein the models are tuned to noise in the training dataset but lack generalizability to external validation datasets [22]. This is especially apparent in Wen et al. [18], who report a near perfect AUC of 0.99 for their best performing model.

Anania et al. [11] reported externally validated performance outcomes, and is the only paper included that does so. However, they introduced a degree of bias via pre‐processing data prior to applying bootstrapping validation, constituting minor data leakage. Capretti et al. [12] did not publish a strategy for how missing data were handled and featured a high risk of bias due to overfitting given a small sample size.

Jones et al. [15], Kovoor et al. [16] and Xu et al. [19] performed internal validation only and as such pose a moderate risk of bias and poor generalizability. Dudurych et al. [13] similarly did not perform external validation, but given the multicentre, international setting from which they sourced data, it is likely that the training dataset offsets the associated risk of bias.

### 3.3 Reported Results

#### 3.3.1 Model Performance

Published performance measures varied across each study and is represented in Table 2. Reported area‐under‐receiver‐operator‐curves (AUROCs) for classifier models ranged from 0.65 [11] to 0.99 [18]. This range narrows to 0.71 to 0.83 when considering studies with a low overall risk of bias, with an averaged AUROC of 0.78 [14, 15]. Regression models uniformly produced poor unstable results. Root mean squared error (RMSE) ranged between 3 [11] to 13 days [17], with less biased papers reporting between 4.6 and 12.7 days [15, 19]. Only Jones et al. [15] and Kovoor et al. [16] included complete model performance values, limiting comparative analyses of model performance.

**TABLE 2:** Model performance.

Study data		Classification metrics				Regression metrics		
Author/Year/Country/Outcome	Algorithm	AUROC	ACC	SPEC	SENS	MAE	RMSE	MAPE
Anania et al. 2024, Italy [11]		0.92	0.94				2.8	0.21
LoS > median (derivation)	Support vector machine	0.86	0.83				5	0.29
	Gradient‐boosting machine	0.81	0.81				4.78	0.38
	GLMNET	0.8	0.81				4.8	0.38
	Linear model	0.78	0.8				4.75	0.38
LoS > median (validation)	Random forest	0.65	0.42				6.04	0.81
	Support vector machine	0.75	0.79				4.68	0.48
	Gradient‐boosting machine	0.67	0.3				6.15	0.91
	GLMNET	0.68	0.43				5.54	0.76
	Linear model	0.67	0.3				6.3	0.93
Capretti et al. 2021, Italy [12]	Logistic regression	0.71		0.63	0.72			
Dudurych et al. 2020, Int [13]	Logistic regression	0.79						
LoS > 14 days	Support vector machine	0.76						
	Artificial neural network	0.79						
Jo et al. 2021, South Korea [14]	XGB/MLP/LR—stomach cancer	0.83	0.81	0.84	0.53			
Prolonged LoS	XGB—breast cancer	0.83	0.82	0.83	0.45			
	MLP—colon cancer	0.71	0.75	0.76	0.26			
Jones et al. 2025, Australia [15]	Random forest	0.78	0.73	0.5	0.85		12.7	
LoS > 7 days (pre‐op)	Logistic regression	0.8	0.72	0.29	0.93			
	XGBoost	0.75	0.7	0.3	0.9			
LoS > 7 days (post‐op)	Random forest	0.8	0.75	0.53	0.86		11.27	
	Logistic regression	0.81	0.74	0.37	0.93			
	XGBoost	0.75	0.72	0.34	0.91			
Kovoor et al. 2023, Australia [16]	Logistic regression	0.72	0.8	0.98	0.09			
Discharge in 12 hours (derivation)	Random forest	0.84	0.84	0.98	0.3			
	XGBoost	0.87	0.84	0.9	0.58			
Discharge in 12 hours (validation)	Logistic regression	0.73	0.81	0.98	0.1			
	Random forest	0.84	0.85	0.98	0.29			
	XGBoost	0.85	0.83	0.89	0.56			
Discharge in 24 hours (derivation)	Logistic regression	0.73	0.78	0.96	0.18			
	Random forest	0.84	0.83	0.97	0.38			
	XGBoost	0.86	0.82	0.89	0.62			
Discharge in 24 hours (validation)	Logistic regression	0.73	0.79	0.95	0.18			
	Random forest	0.83	0.84	0.97	0.36			
	XGBoost	0.85	0.81	0.87	0.6			
Scala et al. 2023, Italy [17]	Multiple linear regression						13	
LoS categorized into groups	Decision tree		0.83		0.88			
	Random forest		0.81		0.88			
	Support vector machine		0.8		0.89			
	Naïve bayes		0.74		0.89			
	MLP		0.74		0.82			
Wen et al. 2023, China [18]	XGBoost	0.92						
LoS > mean	Light gradient boosting	0.95						
	Decision tree	0.92						
	Support vector machine	0.91						
	K‐nearest neighbors	0.9						
	Random forest	0.94						
	Artificial neural network	0.99						
	Deep forest	0.9						
	CatBoost	0.89						
	Logistic regression	0.86						
Xu et al. 2022, USA [19]	LASSO					1.96	4.79	
LoS in days	Random forest					1.88	4.6	
	MLP					2.31	4.94	

*Note:* Table demonstrates extracted performance data for each of the included models. Missing values were metrics that were not included in model reporting. The colours are a heatmap that makes interpreting model performance much easier. Better performance relative to other models is “more green” on the gradient whereas worse performances are “more red”. Each result is mapped to a colour based on its relation to all other results.

Abbreviation: MLP = multilayer perceptron.

#### 3.3.2 Predictor Variable Importance

Five papers published predictor variable importance measures [11, 13, 15, 17, 18]. Of these, four included important predictors that were temporally invalid [11, 13, 17, 18]. These included whether a patient adhered to a fast‐track recovery protocol post‐operatively [11], inclusion of 30‐day readmissions, complications, mortality and pathological specimens [13], day three post‐operative walking distance [18], and the pre‐operative LoS [17].

Consistently important variables reported by Jones et al. [15] included the patient's National Emergency Laparotomy Audit (NELA) score, lactate level, ASA score, and age. Time from admission to intervention was important, but was only applied to the model trained on intra‐operative data [15]. The addition of intra‐operative findings/diagnoses positively impacted model performance for their paper [15].

#### 3.3.3 Algorithms Used

A variety of tree and gradient‐based algorithms were employed (random forest, XGBoost, gradient boosting machines) as well as neural networks. No type appeared to perform consistently better than others. The more complex, resource intensive neural network models did not outperform comparatively simple tree and gradient‐based models.

## 4 Discussion

This review of nine studies applying ML/AI to predict LoS in General Surgery patients demonstrates that model performance varies widely, reporting and analytical standards are inconsistently applied, and a generally high risk of bias undermines the validity of seemingly promising results.

### 4.1 Analysis of Model Performance and Outcome Definitions

Classifier models with a low overall risk of bias display moderate performance with AUROC values ranging between 0.71 and 0.83 [14, 15]. Regressor models produced markedly more varied performance [11, 15, 17, 19]. Heterogeneity in outcome definition across included studies limits pooled analysis of these results, but generally, models that dichotomize patients into long or short admissions outperform those that provide more granular outcomes. Xu et al. [19] achieve the best regression performance through their two‐stage approach of classification and then regression on a narrower subset of patients, thus training the regressor on a less right‐skewed distribution [23].

Kovoor et al.’s [16] approach to predicting timing of discharge through daily reassessment of variables is novel and demonstrated superior performance. When compared with typical LoS prediction models, dynamically predicting a patient's evolving readiness for discharge involves a shorter prediction time horizon. As such, these models are not prone to the compounding uncertainty that is generated throughout a patient's admission and the ceiling of accuracy these models can achieve may be higher than those that seek to predict LoS upfront. A limitation of this approach is that its forecasting potential is restricted by this shorter time horizon. In Kovoor et al.’s [16] model, bed vacancy across a ward or hospital could only be anticipated 12–24 hours in advance. Although dynamic daily prediction improves accuracy, a model capable of reliably estimating LoS in days would provide greater value for hospital‐wide planning, where forecasting needs to extend beyond the next day.

No studies compared their models' predictions to typical LoS estimation methods employed in their centers. Although traditional LoS estimations are likely heterogenous across different centers [2, 24], an attempt to compare proposed ML outputs with human estimators would enable study authors to quantify the difference in performance between these two predictive techniques. This could provide both a benchmark by which ongoing ML and AI performance could be measured, as well as demonstrate credibility for ongoing model development should models outperform human estimators. Given this has not yet occurred, this review is unable to establish whether ML/AI models' estimates are superior to current best practice standards and benchmarks.

Future directions in improving model performance and clinical applicability for predicting LoS should consider a well‐defined, clinically meaningful outcome definition and may benefit from a classification approach with second‐stage regression, as opposed to primary single‐stage regression. Outcome definitions should be considered with regard to the proposed application timing; models seeking to inform individual patient care might benefit from daily reassessment of discharge likelihood within a shortened time horizon, whereas models seeking to forecast ward or hospital‐wide vacancy would likely benefit from LoS prediction in days. Authors should frame their proposed models within a proposed application timing, having considered the decision horizon that they seek to predict LoS for. This could occur in consultation with key stakeholders, such as bed managers, patient flow teams, and ward‐based clinical staff. Models would benefit from being benchmarked against typical LoS estimations, such as multidisciplinary team meeting predictions.

### 4.2 Predictor Variables

Scarcity in reporting predictor variables and their contributions to model outputs limits recognition of actionable insights for future model development. Commonly reported predictors of prolonged LoS in the literature include ASA score, age, operation duration, estimated blood loss and frailty/functional status, and this appears to be corroborated by the included studies that report variable importance [13, 15, 25]. Interestingly, no model included any psychosocial factors as predictor variables, such as at‐home supports, how far the patient lives from the hospital, or the patient's baseline level of independence. LoS is inherently dependent on the healthcare system in which it is measured. Thus, same‐day admission practices, local discharge processes, access to rehabilitation and community services, geographical, social, and cultural factors may influence LoS independently of patients' clinical characteristics. Model validity should be assessed across healthcare systems and their performance should be interpreted with the context of the local health system that they were trained, tested, and applied within. Models as reported by [16] may accommodate this heterogeneity better than the others listed, given they target “discharge readiness” as opposed to actual LoS, which is marginally less impacted by systematic influences. Furthermore, no studies reported a rationale for predictor feature selection. These papers are likely to have benefited from demonstrating key stakeholder involvement in model development and predictor variable selection.

Beyond statistical accuracy, ML/AI models that seek to contribute to clinical care require transparent interpretability, such that their outputs are trusted [26, 27]. Without complete reporting of the variables that inform a model's output, this transparency remains difficult to achieve. This is particularly apparent for neural network‐based models, whose internal representations are not directly interpretable and for which post‐hoc methods of identifying variable importance are approximations [28, 29]. This difficulty should be considered alongside the comparably modest results that neural network models achieved in the reviewed studies; artificial neural networks (ANN) and tree/gradient‐based models, which are easier to interpret, performed similarly to ANNs. This suggests that the increased difficulty required to interpret them makes ANNs less suitable for predicting LoS, at least in the manner that has been demonstrated in the literature to‐date. Future directions for papers reporting on ML/AI models in LoS prediction should include documented strategies for selecting predictor variables as well as demonstrate consideration of the temporal relation of selected variables to the outcome variable. They should also demonstrate predictor variable importance where possible or discuss limitations in model interpretability.

### 4.3 Model Application

The included studies report on models that seek to inform and optimize patient care through the accurate prediction of LoS such that better resource allocation and bed planning can occur. However, many of the studies feature significant methodological flaws or highly biased results that limit the validity of their reported results. As reported previously, key sources of bias across the literature include data leakage arising either from overlap between predictor and outcome variables or from inappropriate pre‐processing, temporal invalidity of predictor variables, limited reporting of performance measures, and application of ML/AI methods without adequate validation, calibration, or transparent reporting. These biases limit the applicability of affected models to external patient cohorts.

A further limitation of the available evidence is the procedure‐specific nature of many of the included studies. No studies were applied to all General Surgery patients without restriction, with all but one [19] limiting their application to procedure or disease‐specific patient populations. This may be appropriate if the models are intended to be applied to restricted sub‐cohorts, but this must be established in reporting of such models, and recognition of their limited generalization to broader heterogenous General Surgery populations is important.

Although low‐bias studies produced modest results, they demonstrate a promising proof of concept and establish a foundation from which future models may evolve. Currently, the low‐bias models are all applied to retrospective datasets. External validation is required to both validate their outputs, as well as to develop further iterations of these models that are generalizable across a wide patient cohort.

### 4.4 Implications for Future Research

The use of ML and AI models could improve LoS prediction for General Surgery patients, but further development and testing is required to ensure validity and applicability to prospective cohorts. Table 3 provides an operational framework for such models that authors could utilize during development to minimize the commonly identified biases in this review. This framework could function as a checklist for authors of LoS‐prediction tools, in both General Surgery applications and beyond. Given the limited and heterogeneous evidence base of nine studies, this framework should be regarded as a preliminary evidence‐informed checklist rather than a formal guideline. The checklist is intended to complement established tools such as PROBAST + AI by translating the recurring limitations identified in this review into practical considerations for model development, validation, reporting, and implementation.

**TABLE 3:** Proposed operational checklist for future LoS prediction studies.

Stage of development	Theme	Guidance
Pre‐training	Scope	Is the model to be used on a hospital‐wide basis, or as a bedside‐tool? Which decision‐making processes are to be assisted? Which decision‐makers will be supported—bed managers/clinicians/patients and their families? Is the model procedure, disease, speciality, or hospital‐wide specific? Is the intended use for elective or emergency patients? What local admission, rehabilitation, and discharge pathways as well as other institutional processes are likely to influence the outcome?
	Features	Which features will inform predictions? How are they selected? Has there been expert and key stakeholder involvement in the selection process? Are the features temporally valid with regard to the target variable? Do any features constitute data leakage?
	Target	How is LoS defined, i.e., classification/regression task, median‐based, or duration remaining prediction? Is it clinically meaningful/linked to resource consumption? Has there been expert and key stakeholder involvement in outcome definition process?
Training and testing	Bias prevention	Ensure that the data has been handled and pre‐processed appropriately as to minimize the risk of introducing bias, i.e., nested imputation/standardization, cross validated testing.
	Benchmarking	Compare model performance to current best practice standards/human estimators of LoS.
	Validation	If possible, externally validate models. If not, ensure adequate cross validation/internal validation has occurred and report reasons preventing external validation.
Reporting	Performance	Report performance metrics in full. Utilize measures specific to classification/regression tasks. Avoid post‐hoc reconfiguration of regression outputs into classification tasks through the use of an “acceptable error margin”, unless clinically meaningful to do so.
	Interpretability	Report, if possible, predictor variable importance. Utilize both model‐specific and agnostic importance metrics, such as SHAP. Describe difficulties in interpretability for neural networks.
	Significance	Propose the clinical use‐cases for models, framed by the timing and type of feature variables required to inform the model as well as the outcome definition. Identify where in the clinical or bed management workflow that the model could best be applied and to what end.

*Note:* Table outlining the key considerations at each step of LoS prediction model development to minimize bias in final development.

## 5 Conclusion

ML/AI models demonstrate promise at predicting LoS for General Surgery patients at risk of prolonged admission with moderate discrimination when study methods are rigorous. Granular regression outputs in days remain unstable and further refinement of models is required to produce clinically meaningful predictions. Sources of bias include heterogenous outcome definitions, mishandled data during pre‐processing, and sparse external validation, all of which limit the generalizability and reliability of study results. Models are currently more credible for operational‐scale forecasting, rather than individual patient forecasting or as a bedside decision‐support tool. Individualized patient‐level forecasting requires further development, validation, and bias‐free reporting. Future studies should seek to standardize definitions of prolonged LoS, restrict predictor variables to prospectively available data, report model performance in full, and undergo external validation.

## Author Contributions

**Joshua Blum:** conceptualization, investigation, writing – original draft, methodology, validation, visualization, writing – review and editing, formal analysis, project administration, data curation, supervision. **Nomiki Glynatsis:** writing – original draft, investigation, methodology, data curation, writing – review and editing. **Brieanna Hill:** investigation, writing – original draft, methodology, writing – review and editing, data curation. **James Montgomery:** conceptualization, writing – review and editing, supervision. **Richard Turner:** conceptualization, writing – review and editing, supervision.

## Funding

The authors have nothing to report.

## Conflicts of Interest

The authors declare no conflicts of interest.

## Data Availability

The data that support the findings of this study are available from the corresponding author upon reasonable request.

## References

[1] A. Singh , P. E. Farmer , J. L. Tully , R. S. Waterman , and R. A. Gabriel , “Forecasting Surgical Bed Utilization: Architectural Design of a Machine Learning Pipeline Incorporating Predicted Length of Stay and Surgical Volume,” Journal of Medical Systems 49, no. 1 (2025): 67, 10.1007/s10916-025-02201-3. PMC12095433 40397217

[2] K. Stone , R. Zwiggelaar , P. Jones , and N. Mac Parthaláin , “A Systematic Review of the Prediction of Hospital Length of Stay: Towards a Unified Framework,” PLOS Digital Health 1, no. 4 (2022): e0000017, 10.1371/journal.pdig.0000017. PMC9931263 36812502

[3] E. F. Philbin and J. B. Roerden , “Longer Hospital Length of Stay Is Not Related to Better Clinical Outcomes in Congestive Heart Failure,” American Journal of Managed Care 3, no. 9 (1997): 1285–1291, https://pubmed.ncbi.nlm.nih.gov/10178477/. 10178477

[4] O. P. Henry , G. Li , R. E. Freundlich , W. S. Sandberg , and J. P. Wanderer , “Understanding the Accuracy of Clinician Provided Estimated Discharge Dates,” Journal of Medical Systems 46, no. 1 (2021): 2, 10.1007/s10916-021-01793-w. PMC8696264 34786607

[5] C. M. Bishop , Pattern Recognition and Machine Learning (Information Science and Statistics) (Springer‐Verlag, 2006).

[6] L. Paina and D. H. Peters , “Understanding Pathways for Scaling up Health Services Through the Lens of Complex Adaptive Systems,” Health Policy and Planning 27, no. 5 (2012): 365–373, 10.1093/heapol/czr054. 21821667

[7] E. Rusoja , D. Haynie , J. Sievers , et al., “Thinking About Complexity in Health: A Systematic Review of the Key Systems Thinking and Complexity Ideas in Health,” Journal of Evaluation in Clinical Practice 24, no. 3 (2018): 600–606, 10.1111/jep.12856. 29380477

[8] P. Chana , M. Joy , N. Casey , et al., “Cohort Analysis of Outcomes in 69 490 Emergency General Surgical Admissions Across an International Benchmarking Collaborative,” BMJ Open 7, no. 3 (2017): e014484, 10.1136/bmjopen-2016-014484. PMC5353261 28274969

[9] K. G. M. Moons , J. A. A. Damen , T. Kaul , et al., “PROBAST+AI: An Updated Quality, Risk of Bias, and Applicability Assessment Tool for Prediction Models Using Regression or Artificial Intelligence Methods,” BMJ 388 (2025): e082505, 10.1136/bmj-2024-082505. PMC11931409 40127903

[10] L. A. McGuinness and J. P. T. Higgins , “Risk‐Of‐Bias Visualization (Robvis): An R Package and Shiny Web App for Visualizing Risk‐of‐Bias Assessments,” Research Synthesis Methods 12, no. 1 (2020): 55–61, 10.1002/jrsm.1411. 32336025

[11] G. Anania , M. Chiozza , E. Pedarzani , et al., “Predicting Postoperative Length of Stay in Patients Undergoing Laparoscopic Right Hemicolectomy for Colon Cancer: A Machine Learning Approach Using SICE (Società Italiana di Chirurgia Endoscopica) CoDIG Data,” Cancers 16, no. 16 (2024): 15, 10.3390/cancers16162857. PMC11352329 39199628

[12] G. Capretti , C. Bonifacio , C. De Palma , et al., “A Machine Learning Risk Model Based on Preoperative Computed Tomography Scan to Predict Postoperative Outcomes After Pancreatoduodenectomy,” Updates Surg 74, no. 1 (2022): 235–243, 10.1007/s13304-021-01174-5. 34596836

[13] I. Dudurych , “Predicting Outcomes of Pelvic Exenteration Using Machine Learning,” Colorectal Disease 22, no. 12 (2020): 1933–1940, 10.1111/codi.15235. 32627312

[14] Y. Y. Jo , J.H. Han , H. W. Park , et al., “Prediction of Prolonged Length of Hospital Stay After Cancer Surgery Using Machine Learning on Electronic Health Records: Retrospective Cross‐Sectional Study,” JMIR Medical Informatics 9, no. 2 (2021): e23147, 10.2196/23147. PMC7939945 33616544

[15] D. Jones , J. Blum , C. Cartwright , et al., “Applying Machine Learning to the ANZELA‐QI Database to Predict Adverse Outcomes for Patients Undergoing Emergency Laparotomy,” ANZ Journal of Surgery 95, no. 10 (2025): 2080‐2087, 10.1111/ans.70185. 40387058

[16] J. G. Kovoor , S. Bacchi , A. K. Gupta , et al., “The Adelaide Score: An Artificial Intelligence Measure of Readiness for Discharge After General Surgery,” ANZ Journal of Surgery 93, no. 9 (2023): 2119–2124, 10.1111/ans.18546. 37264548

[17] A. Scala , T. A. Trunfio , and G. Improta , “Classification and Regression Model to Manage the Hospitalization for Laparoscopic Cholecystectomy,” Scientific Reports 13, no. 1 (2023): 14700, 10.1038/s41598-023-41597-1. PMC10485042 37679406

[18] Z. J. Wen , Y. Wang , S. Chen , et al., “Construction of a Predictive Model for Postoperative Hospitalization Time in Colorectal Cancer Patients Based on Interpretable Machine Learning Algorithm: A Prospective Preliminary Study,” Frontiers in Oncology 14 (2024): 10, 10.3389/fonc.2024.1384931. PMC11211394 38947887

[19] Z. H. Xu , C. Zhao , C. D. Scales Jr , R. Henao , and B. A. Goldstein , “Predicting In‐Hospital Length of Stay: A Two‐Stage Modeling Approach to Account for Highly Skewed Data,” BMC Medical Informatics and Decision Making 22, no. 1 (2022): 12, 10.1186/s12911-022-01855-0. PMC9035272 35462534

[20] G. Anania , F. Agresta , E. Artioli , et al., “Laparoscopic Right Hemicolectomy: The SICE (Società Italiana Di Chirurgia Endoscopica e Nuove Tecnologie) Network Prospective Trial on 1225 Cases Comparing Intra Corporeal Versus Extra Corporeal ileo‐Colic Side‐to‐Side Anastomosis,” Surgical Endoscopy 34, no. 11 (2020): 4788–4800, 10.1007/s00464-019-07255-2. PMC7572335 31741153

[21] M. Kubat , “Addressing the Curse of Imbalanced Training Sets: One‐Sided Selection,” Fourteenth International Conference on Machine Learning (2000), https://www.semanticscholar.org/paper/Addressing‐the‐Curse‐of‐Imbalanced‐Training‐Sets%3A‐Kub%C3%A1t‐Matwin/ebc3914181d76c817f0e35f788b7c4c0f80abb07.

[22] O. Montesinos‐López , A. Montesinos , and J. Crossa , Multivariate Statistical Machine Learning Methods for Genomic Prediction, (2022). 36103587

[23] J. Mullahy , “Specification and Testing of Some Modified Count Data Models,” Journal of Econometrics 33, no. 3 (1986): 341–365, 10.1016/0304-4076(86)90002-3.

[24] D. Allen , “Inside 'Bed Management': Ethnographic Insights From the Vantage Point of UK Hospital Nurses,” Sociology of Health & Illness 37, no. 3 (2015): 370–384, 10.1111/1467-9566.12195. 25524505

[25] T. C. Collins , J. Daley , W. H. Henderson , and S. F. Khuri , “Risk Factors for Prolonged Length of Stay After Major Elective Surgery,” Annals of Surgery 230, no. 2 (1999): 251–259, 10.1097/00000658-199908000-00016. PMC1420868 10450740

[26] A. I. F. Poon and J. J. Y. Sung , “Opening the Black Box of AI‐Medicine,” Journal of Gastroenterology and Hepatology 36, no. 3 (2021): 581–584, 10.1111/jgh.15384. 33709609

[27] M. Ennab and H. McHeick , “Enhancing Interpretability and Accuracy of AI Models in Healthcare: A Comprehensive Review on Challenges and Future Directions,” Front Robot AI 11 (2024): 1444763, 10.3389/frobt.2024.1444763. PMC11638409 39677978

[28] T. Antamis , A. Drosou , T. Vafeiadis , A. Nizamis , D. Ioannidis , and D. Tzovaras , “Interpretability of Deep Neural Networks: A Review of Methods, Classification and Hardware,” Neurocomputing 601 (2024): 128204, 10.1016/j.neucom.2024.128204.

[29] G. Montavon , W. Samek , and K.‐R. Müller , “Methods for Interpreting and Understanding Deep Neural Networks,” Digital Signal Processing 73 (2018): 1–15, 10.1016/j.dsp.2017.10.011.

